# Investigating and preventing scientific misconduct using Benford’s Law

**DOI:** 10.1186/s41073-022-00126-w

**Published:** 2023-04-11

**Authors:** Gregory M. Eckhartt, Graeme D. Ruxton

**Affiliations:** School of Biology, University of St Andrews, St Andrews, KY16 9TH UK

**Keywords:** Scientific misconduct, Peer review, Benford’s Law, Benford’s Law tests, Retracted article testing, Animal behaviour

## Abstract

Integrity and trust in that integrity are fundamental to academic research. However, procedures for monitoring the trustworthiness of research, and for investigating cases where concern about possible data fraud have been raised are not well established. Here we suggest a practical approach for the investigation of work suspected of fraudulent data manipulation using Benford’s Law. This should be of value to both individual peer-reviewers and academic institutions and journals. In this, we draw inspiration from well-established practices of financial auditing. We provide synthesis of the literature on tests of adherence to Benford’s Law, culminating in advice of a single initial test for digits in each position of numerical strings within a dataset. We also recommend further tests which may prove useful in the event that specific hypotheses regarding the nature of data manipulation can be justified. Importantly, our advice differs from the most common current implementations of tests of Benford’s Law. Furthermore, we apply the approach to previously-published data, highlighting the efficacy of these tests in detecting known irregularities. Finally, we discuss the results of these tests, with reference to their strengths and limitations.

**Supplementary Information:**

The online version contains supplementary material available at 10.1186/s41073-022-00126-w.

## Background

Accounts of scientific misconduct can draw widespread attention. Archetypal cases include the study produced by Wakefield et al. [1] linking autism to the vaccine against measles, mumps and rubella, and the decade-long misconduct perpetrated by Diederik Stapel [2, 3]. The problem, however, is far more widespread than often recognised. A meta-analysis of survey data reports that almost 2% of scientists admitted to having fabricated, falsified or modified data on at least one occasion [4]. This is perhaps unsurprising in the context of well-established biases towards the publication of significant results [5–8]; one study suggesting that the likelihood of publishing clinical trial results with statistically significant or positive findings is nearly three times higher than those with non-significant, negative, or perceived-unimportant results [9]. One needs only to look through a list of recent retractions to understand the extent of the issue [10]. The potential consequences of such misconduct are dire, not only in their potential to directly affect human lives, as in the case of unvaccinated children [11], but also in their capacity for reputational damage, to scientists, institutions, fields of research, and the scientific process itself, at a time when societal confidence in published scientific literature has been shaken; with public figures describing scientific data on phenomena such as climate change as “fake news” [12].

The verification of data veracity is a key area of failure in this regard. Currently, consensus regarding efficient methods is lacking. Even in areas of science such as medicine, where the quality of data can be directly linked to human outcomes and monetary gain or loss, guidelines are inconsistent and non-specific in the audit and verification of source data [13]. In many areas of science, peer-review remains the most heavily relied upon means of quality-control in scientific research by journals, whilst academic institutions seem not to focus on prevention or detection, but on investigation only after a whistle has been blown [3]. Although peer-reviewers have undoubtedly become more familiar with the susceptibility of research to misconduct, there has existed little framework to assist in its investigation. Recently, a checklist was proposed which might be used to flag studies which are more vulnerable to manipulation for further investigation [14]. However, it was identified that after screening, there is no clear process which reviewers might be directed to in further investigating research data which they suspect may be fraudulent [14]. We propose that Benford’s Law might provide a useful next step in the investigative process [15]. Analysing the distribution frequency of financial data with reference to Benford’s Law is a well-established fraud analysis technique in the practice of professional auditing [16], and its effectiveness has been shown in detecting fabricated data for example in the fields of anaesthesia, sociology and accounting research ([17–21]; see also [22] where it was not effective for a group of social psychology studies, although we explain later why detection can depend on careful choice of test statistic).

In the present paper we aim to provide a concise set of advice on the implementation of tests of Benford’s Law compliance as a primer for those wishing to further investigate data highlighted as problematic, of value to investigations involving routine monitoring as part of the peer-review process, or those targeted at specific work where concern has been raised. This builds on the seminal works of, for example Diekmann [21], by synthesising the available literature and providing useful conclusions based on the weight of evidence presented. We discuss the qualities a sample of data might have that make it more or less likely to conform to Benford’s Law, and offer guidance on ways to test for adherence to Benford’s Law statistically. We then take an example from animal personality data to explore the test’s effectiveness with real data in a field to which it has not been previously applied and explore how statistical testing can be augmented by the use of comparator data-sets that are not under suspicion. Ultimately, we thus aim to contribute to the conception of an overall framework which investigators might refer to in the inspection of potentially fraudulent research.

## Identifying abnormal patterns in data

**Fig. 1:**
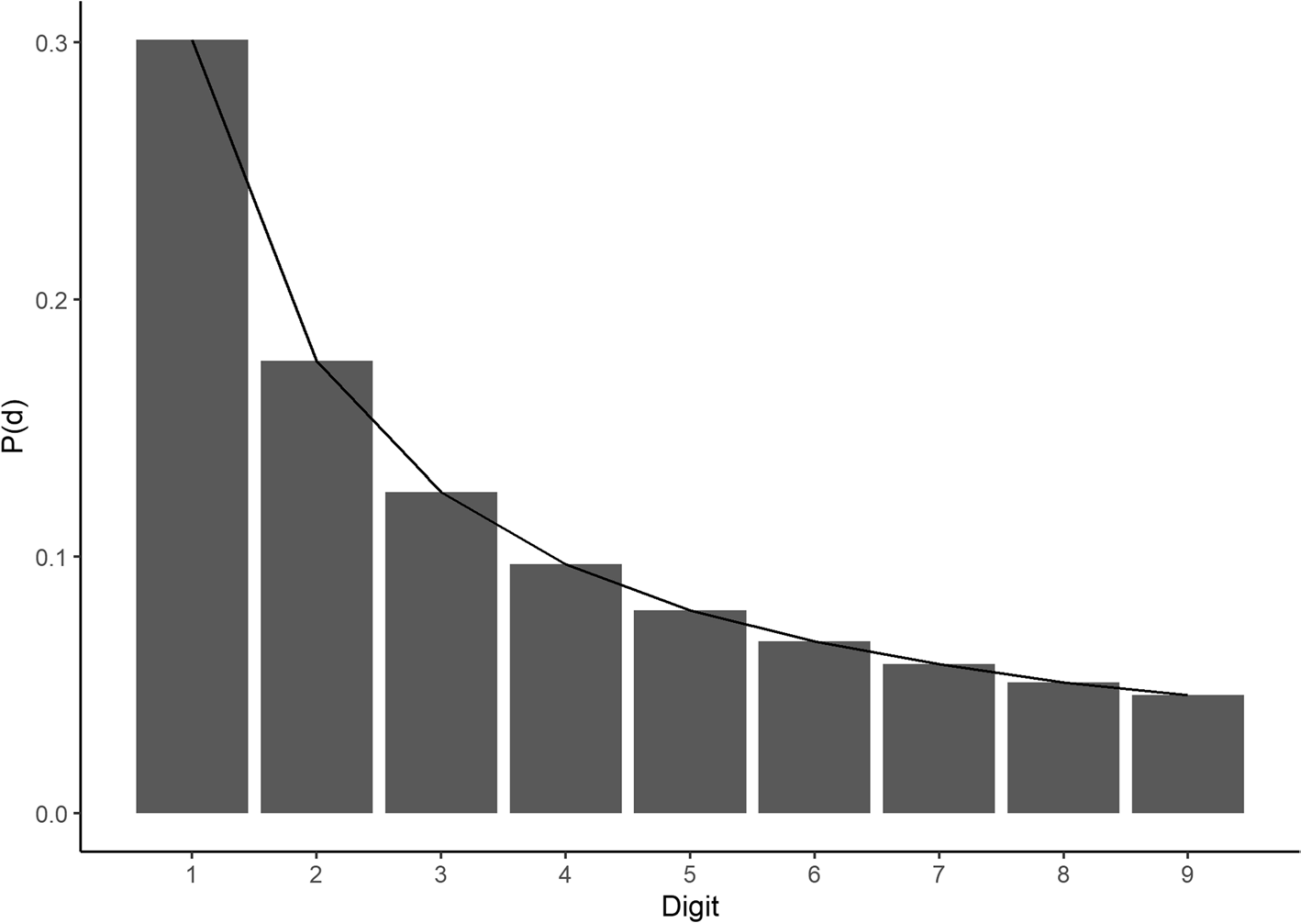
Benford’s Law for the first digit**.** Graphical depiction of Benford’s Law as applied to the first digits of a notional dataset that perfectly fits the law, displaying the characteristic negative logarithmic curve of occurrence probability, *P(d)*, as the digit value increases

Benford’s Law is a well-established observation that, in many numerical datasets, a distribution of first and higher order digits of numerical strings has a characteristic pattern. The observation is named after the physicist Frank Benford [15] who reported it in a paper regarding “The Law of Anomalous Numbers”, although it was actually first stated by Simon Newcomb [23] and is sometimes referred to as the Newcomb-Benford Law. In its light version, it states that the first digit, *d*, of numerical strings in datasets that follow this distribution is more likely to be 1 than any other value, with decreasing probability, *P(d)*, of the digit occurrence as it increases in value (see Eq. 1 below and Fig. 1). This phenomenon can be observed across a wide array of datasets, including natural data such as global infectious disease cases and earthquake depths [24], financial data [25], genome data [26], and mathematical and physical constants [15].

1 $$P\left(\text{d}\left|i\right.\right)=log_{10}\left(1+\frac1d\right)$$

where *i* = 1 and 1 ≤ *d* ≤ 9

Furthermore, the law can be generalised to digits beyond the first, such that we can predict the probability of occurrence, *P(d)*, of any digit, *d*, in any position, *i*, within a given string using the conditional probabilities of the preceding digits ([27]; see Table 1 and Eq. 1 (for *i* = 1) & 2 (for *i* > 1)). This can be especially important in assessing adherence to a Benford’s Law distribution, as data fabricators will often neglect to conform digits subsequent to the first to any kind of natural distribution [21].

2 $$P\left(\text{d}\left|i\right.\right)=\sum_{k=10^{i-2}}^{10^{i-1_{-1}}}log_{10}\left(1+\frac1{10k+d}\right)$$

**Table 1:** Probability of digit *d* in position *i* of a numerical string per Benford’s Law for the first 4 digits

Digit, ***d***	Position***, i*** = 1	***i*** = 2	***i*** = 3	***i*** = 4
0		0.11968	0.10178	0.10018
1	0.30103	0.11389	0.10138	0.10014
2	0.17609	0.10882	0.10097	0.10010
3	0.12494	0.10433	0.10057	0.10006
4	0.09691	0.10031	0.10018	0.10002
5	0.07918	0.09668	0.09979	0.09998
6	0.06695	0.09337	0.09940	0.09994
7	0.05799	0.09035	0.09902	0.09990
8	0.05115	0.08757	0.09864	0.09986
9	0.04576	0.08500	0.09827	0.09982
**E(d)**	3.43973	4.18712	4.46773	4.49677
**σ**^**2**^_**d**_	6.05554	8.25399	8.25120	8.25112

Where *i* > 1

Deviations from Benford’s Law then, in datasets where we expect to see adherence to this digit distribution, can raise suspicion regarding data quality. Indeed, financial auditors have been using Benford’s Law for some years to test datasets’ adherence to the expected distribution in order to detect possible fraudulent manipulation [16]. It has also been applied recently in the analysis of COVID-19 data and the potential spuriousness of some countries’ self-reported disease cases [28, 29]. Accordingly, it has been suggested that Benford’s Law provides a suitable framework against which scientific research data can be inspected for possible indications of manipulation [21, 30].

In order to do so, we must first define datasets which are appropriate for this use and for which we would expect to see adherence to BL. In general, it is expected that datasets where individual values span multiple orders of magnitude are more likely to abide by BL. There is no set minimum number of datapoints, although a good rule of thumb can be derived from a power analysis by Hassler and Hosseinkouchack [31], that generally the statistical tests for deviations from Benford’s Law will be most effective with at least *N* ≥ 200. However, even for sample sizes as small as 20, some testing may be worthwhile (see [32] for approaches in this case).

This assumption being satisfied, we should more specifically expect data with a positively skewed distribution, as is common in naturally occurring data (such as river lengths or fishery catch counts), to adhere to BL. This includes such distributions as the exponential, log-logistic, and gamma distributions [33]. Furthermore, we can expect figures derived from combinations or functions of numbers such as financial debtors balances, where price is multiplied by a quantity [34], or the regression coefficients of papers within a journal [21], to conform with Benford’s Law. Note that this should be true irrespective of the unit of measurement, i.e. the distribution of digits should be scale invariant [27].

There are also some cases where we might expect digits following the first, but not the first digit of some data to follow Benford’s Law. For example, stock market indexes such as the FTSE 100 over time, for which the magnitudes of the first digits are constrained (having never exceeded 8000 at the time of writing) but for which the subsequent digits do follow the expected Benford’s Law distribution reasonably closely.

Equally, there are many datasets for which a Benford’s Law digit distribution may not be appropriate. This is true of data that is normally or uniformly distributed. The Benford’s Law digit distribution should also be expected not to be met by data that is human-derived to the extent that no natural variation would be expected, such as prices of consumer goods, or artificially selected dependent variables such as the volume of a drug assigned to different treatment groups [33, 34]. Ultimately, the reviewer must apply professional judgement and scepticism in choosing appropriate datasets for analysis by reference to a Benford distribution. Implicit in this is the requirement that investigators determine and justify whether data should be expected to conform to Benford’s Law prior to any testing of that conformity. Table 2 provides a non-exhaustive summary of properties of appropriate and inappropriate data for Benford analysis.

**Table 2:** Appropriate data for Benford analysis

**Likely Appropriate Data**	**Examples**
*Datasets spanning several orders of magnitude*	*World country populations across time*
*Data derived from natural phenomena*	*Mathematical and physical constants*
*Data with a positively skewed distribution, where the mean is greater than the median*	*Much ecological data such as river lengths*
*Sets of numbers derived from combinations or functions of numbers*	*Regression coefficients of papers within a journal*
**Likely Inappropriate Data**	**Examples**
*Sets of assigned numbers or those driven more by human than natural processes*	*Sample or participant identification numbers, house prices*
*Data that does not span several orders of magnitude (although we may apply the law to subsequent digits to the first)*	*Human heights, some stock market indexes*
*Data with an expected specific non-positively skewed distribution*	*Binomial survival probability of polar bears across seasons*

Once an appropriate dataset has been selected, we may assess conformance to Benford’s Law in a number of ways. There are several options to choose from in testing adherence to Benford’s Law statistically. Goodness-of-fit tests, including for example Cramér–von Mises, Kolmogorov-Smirnov, or Pearson’s 𝜒^2^-test, might seem most appropriate, and indeed seem to be the most often used tests in the Benford’s Law literature [31]. Determining the best test is not as simple as it may appear however, with consideration of sensitivity to different types of deviation from the law, avoidance of mistakenly suggesting deviation where none exists, interpretability and parsimony.

Hassler and Hosseinkouchack [31] conducted power analysis by Monte-Carlo simulation of several statistical tests of adherence to Benford’s Law using various sample sizes up to *N* = 1000, including Kuiper’s variant of the Kolmogorov-Smirnov test, Cramér–von Mises, Pearson’s 𝜒^2^-test with 8 degrees of freedom (9 for *i* > 1), (Eq. 3 below), and a variance ratio test developed by the authors [35]. They found all of these tests to be underpowered at detecting the types of departure investigated in comparison to the simple 𝜒^2^-test with one degree of freedom suggested by [36], (Eq. 4), which compares the mean of the observed frequency of *d* to that of the expected frequency. They recommend further, that for Benford’s Law for the first digit, greater power can be achieved by a one-sided mean test ‘Ζ’, (Eq. 5), if one can justify the a priori assumption that the alternative hypothesis is unidirectional. This may be assumed if we believe a naïve data fabricator might tend to fabricate data with first digit probabilities closer to a uniform distribution, biasing the probability of higher-order digits in the first position, thus increasing the mean, $\overline{d}$, of the observed first digits in comparison to the expected mean, *E(d)* (see a summary of *E*(*d*) in Table 1); although see Diekmann [21] who suggests that fabricators may intuitively form a reasonable distribution of first but not second digits. Accordingly, the null hypothesis in Ζ is rejected where $\overline{d}>E(d).$

What we refer to as the 𝜒^2^-test with 8 or 9 degrees of freedom, the 𝜒^2^-test with one degree of freedom and the Z test, respectively, have calculated values as defined below:

3 $${\textrm{X}}_{8\kern0.24em or\kern0.24em 9}^2=N\sum \limits_{d=1\kern0.24em or\;0}^9\frac{{\left({h}_d-{p}_d\right)}^2}{p_d}$$

4 $${\textrm{X}}_1^2=N\frac{{\left(\overline{d}-E(d)\right)}^2}{{\sigma^2}_d}$$

5 $${\displaystyle \begin{array}{c}\textrm{Z}=\sqrt{N}\frac{\overline{d}-E(d)}{\sigma_d}\\ {}{H}_1:\overline{d}>E(d)\end{array}}$$

Where:

*N* is the number of observed digits

*d* is an index for each possible digit

*h*_*d*_ is the observed frequency of digit *d* (such that the sum of these frequencies adds up to 1)

*p*_*d*_ is the expected frequency of digit *d* (see Table 1)

$\overline{d}$ is the mean of the *N* observed digits ($\overline{d}={N}^{-1}\sum_{j=1}^N{d}_j$) and *d*_*j*_ is the observed digit value at the relevant position corresponding to datapoint *j* of the dataset of N observed digits, where 1 ≤ *j* ≤ *N*.

*E(d)* is the expected digit mean (see Table 1)

*σ*_*d*_ is the standard deviation of expected digits (see Table 1)

Further simulations can be seen in Wong [37], using greater sample sizes, suggesting, in the absence of the variance ratio and 𝜒^2^-test with one degree of freedom tested in Hassler and Hosseinkouchack [31], that Cramer von-Mises or Anderson-Darling tests can provide the greatest power to detect some types of deviation. More importantly however, Wong [37], having simulated with greater sample sizes, suggests that with increasing sample sizes (N > ~ 3000), the rejection rate of the null hypothesis, in any such test, increases significantly, even for distributions that deviate only very slightly from the null distribution.

With consideration to statistical power, complexity, interpretability, and parsimony, we therefore recommend that Pearson’s 𝜒^2^-test with one degree of freedom, Eq. 4, provides an effective overall test statistic for the adherence to Benford’s Law of an appropriate dataset. Furthermore, when testing for adherence to Benford’s Law for the first digit only, we echo the sentiments of Hassler and Hosseinkouchack [31], that it may be appropriate to increase the power of the test by assuming a unidirectional alternative hypothesis and applying a one-tailed variant of the test. Of course, investigators may often want to utilise multiple tests. Indeed, there is reason in some cases to argue that the tests of digit means in Eqs. 4 & 5 are less informative than the chi-squared test in Eq. 3. These tests are useful as a first port of call when testing general hypotheses regarding the distribution of fabricated digits, however they are on odd occasions less sensitive than Eq. 3 to substantial variations in individual digits. For example, if we believe that a fabricator might produce an overabundance of fives and zeros in the second position of numerical strings than is expected naturally, Eqs. 4 & 5 may not detect this if the mean value of digits in this position are compensated by the distribution of the other digits. In such a situation it is of value to adopt a further statistic, and the chi-square test in Eq. 3 is generally a useful option.

It is important to note that statistically significant deviations from Benford’s Law need not be caused by fraudulent manipulation, as typified by the suggestion of Wong [37], that greater and greater sample sizes will increase the likelihood very small deviations from the null distribution being detected. Also testing multiple digit positions within the same data-set will increase the chance of type I error. This should be acknowledged, or controlled for using a procedure like Bonferroni correction, or a compound test across multiple digits used (see [32] for useful approaches in this regard). Data irregularities may also arise as a result of error rather than manipulation. Even with the most parsimonious test, caution and forethought must be applied in the use of such tests with certain datasets. We recommend plotting the expected and observed distributions of digits as an intuitive means of estimating the strength of any deviation from the expected distribution. A reusable code snippet has been provided in the additional file (part 1. Reusable Benford’s Law tests and graphs) which may be used to extract digits from numerical strings in a dataset, plot the associated distributions, and apply the tests under Eqs. 3 to 5. Investigators may also prefer to use the benford.analysis package for plotting [38].

Whilst it is provable mathematically that a scale-neutral, random sample of numbers selected from a set of random probability distributions will follow Benford’s Law [27], Benford’s Law is not immutable or irrefutable for real data. Whilst we can observe that Benford’s Law holds remarkably well for certain datasets, reflecting Hill’s theoretical proof and the idea that such data is ultimately the product of random processes and random sampling, in reality we know that no such dataset is truly completely random in its construction or sampling. As such, we can expect minor deviations from Benford’s Law even in datasets which fit all of the supposed criteria for suitable data. Thus, it is not possible to prove unquestioningly that some set of data should, or should not, follow an exact distribution such as Benford’s Law. Justification for expecting a given data set to conform to Benford’s Law can come from discussion of the criteria already mentioned, but also from demonstrated conformity to Benford’s Law of similar independently-obtained datasets of similar data. Thus, we suggest that investigations of a suspect dataset through exploration of adherence to Benford’s Law will be greatly strengthened if appropriate “control” datasets are subject to the same testing. This we put to the test in the following section, "Application to real data". Clearly, ideally the person carrying out such testing should be blind to which datasets are controls and which are the focal suspect ones.

## Application to real data

In order to sufficiently demonstrate the efficacy of the described approach, we have applied the test of conformity to Benford’s Law to a number of existing publicly available datasets. First, we applied the test to datasets from publications which were retracted for suspected irregularities in the data. We then compared this to similar datasets with no such retractions or public suspicions of data abnormalities, to assess whether and when the test does or does not detect known irregularities.

## Methods

First, we sought to identify published articles with data which is likely to contain irregularities. We used Retraction Watch Database [10] to search for retracted research articles tagged with expressions of concern about the underlying data. We limited this search to articles published by ‘Royal Society Publishing’, which has implemented a strong open data policy since 2013 [39, 40]. It is perhaps unsurprising that it is otherwise exceedingly difficult to find publicly available data from publications which have been retracted for data issues. The exact search criteria used can be found in the additional file (part 2. Searches and methods). We manually scanned each of the 23 items identified by this search (some of which were duplicates of the same article with different levels of notice), identifying two articles which met all of the criteria for testing, including publicly available and practically useable data, suitability for Benford’s Law analysis, and retraction for issues in the underlying data (henceforth, articles 1 & 2, see Table 3). The conclusions of both studies generally rely on data concerning individual differences within consistent aspects of animal behaviour, or ‘personality’ as it is often referred to [46]. This is a natural phenomenon which is well-researched within behavioural ecology and generally understood to be the result of natural processes of genetic expression and environment. Data resulting from many methods of personality measurement, such as the time for a fish to emerge from a refuge after being placed in a novel site (e.g. [45, 47]), are found to have distributions across populations which mimic that of other natural processes, being positively skewed [48] and thus conforming to the criteria outlined in "Identifying abnormal patterns in data" (see Table 2).

**Table 3:** Results of the main tests of adherence to Benford’s Law for five datasets of animal personality data (Figs. 2 and 3)

Article	Position, *i*	N	${\upchi}_1^2$	Ζ	${\upchi}_{8\ or\ 9}^2$	Reference	Figure
1	1	1063	12.53***	−1.44	251.08***	[41]	2 (top)
1	2	1063	9.64***		83.12***	[41]	2 (top)
2	1	1436	27.50***	−2.13	297.06***	[42]	2 (bottom)
2	2	1436	25.15***		60.89***	[42]	2 (bottom)
3	1	164	0.47	0.28	18.90*	[43]	3 (top)
3	2	164	0.24		9.45	[43]	3 (top)
4	1	316	0.93	−0.39	8.41	[44]	3 (middle)
4	2	316	0.10		11.29	[44]	3 (middle)
5	1	106	0.18	− 0.17	9.05	[45]	3 (bottom)
5	2	106	2.91		16.07	[45]	3 (bottom)

*p < 0.05

**p < 0.01

***p < 0.001

**Fig. 2:**
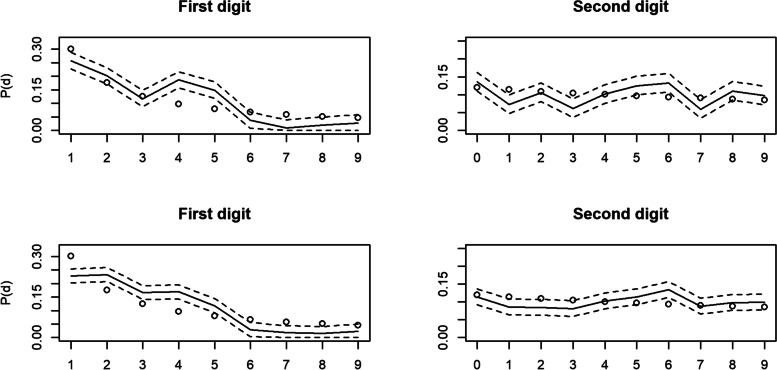
Benford’s Law tests for articles 1 & 2. Distribution of digit value frequencies for the 1st (left panels) and second (right panels) digit positions of data from datasets of animal personality measures, taken from research articles retracted for suspicions of data fabrication, with 95% Sison & Glaz confidence intervals represented by the dashed lines. Dots represent the Benford expected frequency of digits, whilst the solid line represents the observed frequency. Top 2: Article 1. Bottom 2: Article 2

**Fig. 3:**
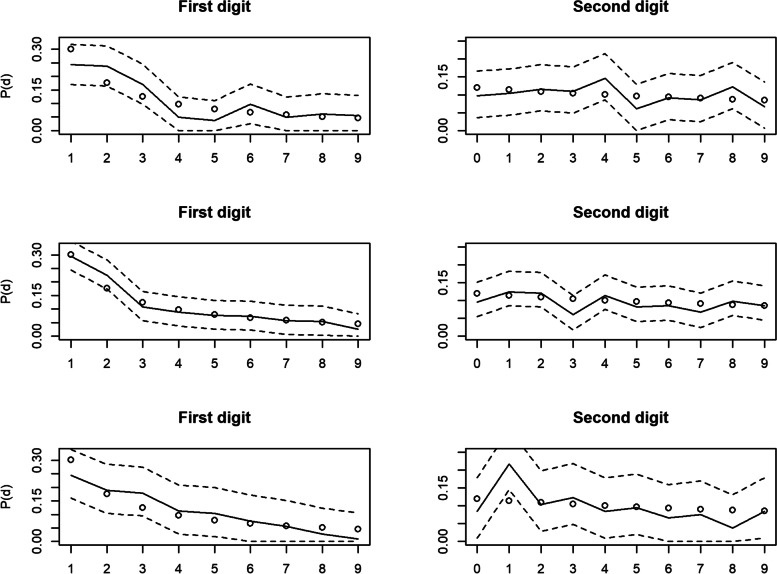
Benford’s Law tests for articles 3 to 5. Distribution of digit value frequencies for the 1st (left panels) and second (right panels) digit positions of data from datasets of animal personality measures, taken from research articles not retracted for suspicions of data fabrication, with 95% Sison & Glaz confidence intervals represented by the dashed lines. Dots represent the Benford expected frequency of digits, whilst the solid line represents the observed frequency. Top 2: Article 3. Middle 2: Article 4. Bottom 2: Article 5

Next, we sought to identify research articles with data on the same type of phenomena, which were as similar as possible to articles 1 & 2, but differentiated by having no notices of retraction or public suspicions of irregularities within the data. To achieve this, we identified the general topic of the two retracted articles and created a search directly within the Royal Society Publishing website’s journal search tool. This centred around research articles with titles containing the words ‘personality’, ‘boldness’ or ‘bold’, published from 2014 onwards (as in the previous search). The search criteria and results can be found in the additional file (part 2. Searches and methods). The first 30 publication results were manually scanned for appropriate data. Eight publications were deemed to be appropriate based on methodological similarities with articles 1 & 2, conformance with the criteria in "Identifying abnormal patterns in data", and the availability and useability of the underlying data.

For methodological convenience, such studies of personality often constrain the maximum values of time to emerge/ resume activity, assigning a maximum value where the animal is found not to have emerged/ resumed activity after a specified length of time. In this way, the associated data contain several experimenter-assigned numbers, which artificially skew the data and inflate the number of zeros in the digits subsequent to the first of numerical strings within the data. In general Benfords Law is not expected to apply (at least to the first digit) when data contains an imposed maximum and/or minimum value. As such, we only analyse a subset of the data for these sets, being all data with values less than the artificial maximum value assigned by the authors. Accordingly, we sought to test datasets with the highest levels of useable data for Benford analysis. Data was therefore required to be as numerous as possible to maximise the power of analysis, whilst maximising the available orders of magnitude, being those datasets with the greatest artificially-assigned maximum. In the absence of a strong argument to favour either criterion, we chose to rank each of the eight studies according to the two criteria with equal weighting. In this way, we could empirically determine the three studies with the highest combined rank for testing (articles 3 to 5, see Table 3 and the additional file; part 2. Searches and methods). For consistency, only time data on personality was assayed across all five datasets.

For each of the datasets identified in accordance with the criteria above then, we used R version 4.0.4 to extract the digits from the numerical strings of each datapoint to ascertain the distribution frequency of digits in the first and second positions. Using those distribution frequencies, we were able to visualise conformity with Benford’s Law and estimate the goodness-of-fit using chi-squared and Ζ tests in accordance with Eqs. 3–5 outlined in "Identifying abnormal patterns in data". Simultaneous confidence intervals were estimated and graphed for each set of digits using the method of Sison and Glaz [49], which can account for multinomial proportions, employing the R package MultinomialCI [50]. The code employed in analysing these datasets is available in the OSF repository [51]. This would easily be modified for readers interested in conducting similar analyses. In this regard there is also a useful R package benford.analysis [38].

## Results

Under ${\upchi}_1^2$, articles 1 & 2 deviated significantly from Benford’s Law for digits in the first and second positions, whilst they did not deviate significantly for articles 3 to 5 (summarised in Table 3). Under Ζ, none of the articles deviated significantly for first position digits. This is due to $\overline{d}<E(d)$ in both instances, thus rejecting the null hypothesis. Finally, under ${\upchi}_{8\ or\ 9}^2$, articles 1, 2 & 3 deviated significantly from Benford’s Law for digits in the first position. However, for digits in the second position, only articles 1 & 2 deviated significantly from Benford’s Law.

As can be seen in Table 3, for both 1st and 2nd digits, ${\upchi}_1^2$ raised concerns about the data in the two articles that had already been identified as problematic, but never for the three comparator datasets. Conversely, Ζ raised no concerns about any of the articles, and ${\upchi}_8^2$ raised concerns about the two “problematic” articles, but also suggested a possible “false positive” concern about article 3.

### Discussion

Generally, the present results build on the growing evidence base indicating that Benford’s Law is an effective means of screening data for potential fabrication (e.g. [21, 30, 52]). Furthermore, the results of this study highlight the importance of understanding the data that one is investigating, as well as the limitations and advantages of different tests of adherence to Benford's Law. For example, although the chi-squared test with one degree of freedom (Eq. 4) performed well using the distribution of first digits to flag data which was known to contain issues, the variant of the chi-squared test under 8 or 9 degrees of freedom (Eq. 3) did not, while the one-sided Z-test (Eq. 5) proved insensitive. We therefore reiterate our earlier statement that Eq. 4 is a useful tool for initial screening whilst Eqs. 3 & 5, together with exploratory visual analysis of graphs, can be useful in testing specific hypotheses regarding the nature of potential data fabrication. Indeed, visual analysis of the graph of observed first digits from article 3 reveal little concern despite the possible “false positive” indicated by Eq. 3.

Given that the data does not span more than 3 to 4 orders of magnitude, one might argue that tests for digits in the first position inflate the likelihood of error compared with digits in the second position. In the case of the Z-test and chi-squared test with 1 degree of freedom, this means that it is difficult to justify the assumption that the expected digit mean might resemble that of Benford's Law. In this case, it is of comfort that we are able to apply the model to digits beyond the first, where the distributions of digits are less affected by orders of magnitude. Indeed, the “false positive” identified here builds on the “false negative” findings by Diekmann [21], illustrating that Benford’s Law tests are often more effective at flagging data issues using the distribution of second and higher digits [21].

Consistent with the published notices of retraction to articles 1 & 2 [53, 54], the tests employed in the present study flagged issues in the data which, upon closer inspection, contained inexplicable duplications. In the case of both articles, retractions were issued just less than 6 years after the publication of the original articles. It is argued that the journals might have much more quickly detected this error using the tests employed in the present study, and in so doing have protected their reputations, and the integrity of scientific literature more generally. With this being said, it is commendable to have required the public availability of source data in the first place, without which such scrutiny and re-examination would not be possible. We argue that scientific integrity would be improved immeasurably by the standardisation of such requirements upon publication. Furthermore, we argue that the use of statistical tests such as those outlined here provide a useful foundation on which to build a framework for the prevention and detection of scientific misconduct through the manipulation of data, which might be used by individual peer-reviewers, academic journals, and scientific institutions alike. However, given the risk of any statistical test of false positives (and negatives), statistical testing can only be a part (albeit a valuable one) of investigating potential fraud.

It is important to note, that fraudulent data manipulation may manifest in ways that are less detectable by analyses of adherence to BL, or be present in data that is not appropriate for such analyses as they would not be expected to adhere to BL. It is of comfort therefore, that the statistical toolbox for investigators is vast, given the appropriate expertise. For example, an investigator might test the hypothesis that a researcher has fabricated clinical trial data for two supposedly randomised trial groups by assessing the under- or over-dispersion of the summary statistics. Indeed Barnett [55] provides a comprehensive analysis of such a test’s effectiveness, concluding that it can be a useful flag of suspect clinical trials in targeted checks. It might reasonably also be applied to the statistics of other between-groups experimental data. The consideration of a broad range of statistical tests will be of great import in the journey towards a framework for the detection and prevention of scientific misconduct. Recent work has demonstrated in the context of international trade data, how we might identify features of data for which Benford’s Law should hold in the absence of fraudulent data manipulation, how application of the law can be modified where conformity cannot be expected, and how evidence of such fraudulent activity can be gathered in this context [56]. Exploration of the applicability of these findings to other areas of potential data manipulation would further valuably expand our detection toolkit. Similarly, recent work [32] has suggested that testing procedures that use a combination of existing tests can be very effective at detecting departures from Benford’s Law even for datasets with as few as 20 datapoints. Further exploration of these approaches, perhaps in exploring their performance on datasets already considered a matter of concern, like the approach taken here, would be very valuable.

## Conclusions

It is of consummate importance that confidence in science is maintained. In providing a unified approach by which reviewers might investigate suspect data, and empirically validating its efficacy, it is hoped that we have suggested the potential to improve the assurance we gain over scientific data. It remains a significant issue that the controls over source data in scientific literature are clearly not sufficient. It is hoped however, that in describing a practical approach, academic institutions and publishers might consider some level of reform or improvement in the controls employed in preventing and detecting scientific misconduct. Heightened rigour in the scrutiny of scientific research is inevitable. Ultimately, the leaders and first-adopters in this field would be rewarded by mitigating their risk of association with fraudsters, and contributing to the ethical maintenance of truth in science.

## Supplementary Information

**Additional file 1.**

## Data Availability

The datasets analysed during the current study are publicly available in the repositories associated with [41–45]. The code employed in analysing these datasets is available in the OSF repository [51]. The results and criteria of the searches employed in the "Methods" section are also available as the additional file part 2. The data analysed in the additional file part 1 is available in the World Bank Group repository, https://data.worldbank.org/ [57].
