# Supplementary material for: Investigating and preventing scientific misconduct using Benford’s Law
Source: Res Integr Peer Rev. 2023 Apr 11;8:1. doi: 10.1186/s41073-022-00126-w (PMC10088595; doi:10.1186/s41073-022-00126-w)
Supplement: Supplementary file 1 — Additional file 1. [file 41073_2022_126_MOESM1_ESM.pdf]

Online Supplement

Table of Contents

1. Reusable Benford's Law tests and graphs **2**

2. Searches and methods **8**

Appropriate RSP papers 8

RSP search 10

RWD search 17

Reusable Benford's Law tests and graphs

Load and prepare your data set

```
dat.pop <- read.csv("WorldBank Population Data.csv", header = T) #example dat
aset from
#https://data.worldbank.org/indicator/SP.POP.TOTL?most_recent_value_desc=true
#https://data.worldbank.org/summary-terms-of-use
#at 17/11/21

#transform data you want to test to long form data

library(reshape2)
dat.pop <- melt(dat.pop, value.name = "population.1000s") #adjust as appropri
ate for your data

#set your parameters

dat.pop <- subset(dat.pop, population.1000s > 0) #remove NAs

dat <- dat.pop$population.1000s #set the vector or timeseries of data you are
testing

str(dat) #check number of datapoints

hist(dat) #check data distribution

x <- 16123 #change to number of datapoints in your timeseries or vector
```

Extract digits and establish cumulative distribution frequencies

```
spcfc.dgt <- function(x, n, data){ #function for taking a specific digit from
a
                               #numeric datapoint within a time-series or vec
tor
if (is.na(as.numeric(substr(as.character(data[x]),n,n))==TRUE)==TRUE) {
  as.numeric(substr(as.character(data[x]),n+1,n+1))
}
  else  {as.numeric(substr(as.character(data[x]),n,n))}
}

DigCDF <- function(n){ #function for cumulative distribution frequency of dig
its
                     #in position 'n'
  Dgts<-c(rep(0,10))
  for(d in c(0,1,2,3,4,5,6,7,8,9)){
    Dgts[d+1] <- sum(log10(1+(1/(10*((10^(n-2)):(10^(n-1)-1))+d))))
  }
```

```
  if(n==1){
    return(c(0,Dgts[1:9]))
  }
  else{
    return(Dgts)
  }
}

#benford digit 1, 2 and 3 probabilities

p1.benford <- DigCDF(1)
p2.benford <- DigCDF(2)
p3.benford <- DigCDF(3)

#code for benfords law test for leading digits

D1 <- c(1:x)
for(d in 1:x){
    D1[d] <- spcfc.dgt(d,1,dat)
}

P1 <- c(0,table(D1)/x)

#code for benfords law test for digits beyond the first

n <- 2 #change to nth digit you are looking at
D2 <- c(1:x)

for(d in 1:x){
  D2[d] <- spcfc.dgt(d,n,dat)
}

P2 <- c(table(D2)/x)


#third digit

n <- 3 #change to nth digit you are looking at
D3 <- c(1:x)

for(d in 1:x){
  D3[d] <- spcfc.dgt(d,n,dat)
}

P3 <- c(table(D3)/x)
```

Plot

```
#extract multinomial 'Sison' 95% CIs (Sison & Glaz, 1995) using MultinomialCI
#package (Villacorta, 2021)

library(MultinomialCI)
SisonD1<-multinomialCI(table(D1),0.05)
SisonD2<-multinomialCI(table(D2),0.05)
SisonD3<-multinomialCI(table(D3),0.05)

#############################################################################
##

par(mfrow=c(2,2))

##plot D1 with CIs

plot(c(0:9),p1.benford,xlab="D",ylab="P",ylim=c(0,0.33))
lines(0:9,P1)
lines(c(0:9),c(0,SisonD1[,1]),lty='dashed')
lines(c(0:9),c(0,SisonD1[,2]),lty='dashed')

##plot D2 with CIs

plot(c(0:9),p2.benford,xlab="D",ylab="P",ylim=c(0.08,0.15))
lines(0:9,P2)
lines(c(0:9),SisonD2[,1],lty='dashed')
lines(c(0:9),SisonD2[,2],lty='dashed')

##plot D3 with CIs

plot(c(0:9),p3.benford,xlab="D",ylab="P",ylim=c(0.085,0.11))
lines(0:9,P3)
lines(c(0:9),SisonD3[,1],lty='dashed')
lines(c(0:9),SisonD3[,2],lty='dashed')
```

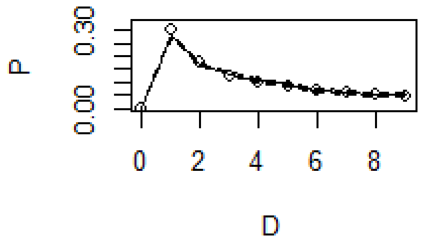

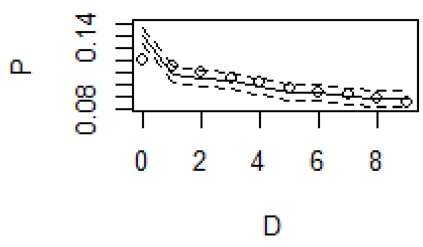

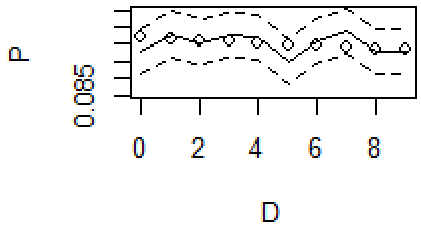

Test goodness of fit

```
#Pearson's Chi-square goodness-of-fit test

chisq.test(table(D1),p = p1.benford[2:10])

##
##  Chi-squared test for given probabilities
##
## data:  table(D1)
## X-squared = 90.195, df = 8, p-value = 4.245e-16

chisq.test(table(D2),p = p2.benford)

##
##  Chi-squared test for given probabilities
##
## data:  table(D2)
## X-squared = 64.266, df = 9, p-value = 2.004e-10

chisq.test(table(D3),p = p3.benford)

##
##  Chi-squared test for given probabilities
##
## data:  table(D3)
## X-squared = 11.964, df = 9, p-value = 0.2153
```

```
############################################################################
##

#chi-square test 1df and table of E(d) & Var(d) for first 3 digits

benfordVector<-function(d){
  c(rep(0,DigCDF(d)[1]*x),rep(1,DigCDF(d)[2]*x),rep(2,DigCDF(d)[3]*x),
    rep(3,DigCDF(d)[4]*x),rep(4,DigCDF(d)[5]*x),rep(5,DigCDF(d)[6]*x),
    rep(6,DigCDF(d)[7]*x),rep(7,DigCDF(d)[8]*x),rep(8,DigCDF(d)[9]*x),
    rep(9,DigCDF(d)[10]*x))
}

benfordMeanD1 <- mean(benfordVector(1))
benfordVarofD1 <- sd(benfordVector(1))^2

benfordMeanD2 <- mean(benfordVector(2))
benfordVarofD2 <- sd(benfordVector(2))^2

benfordMeanD3 <- mean(benfordVector(3))
benfordVarofD3 <- sd(benfordVector(3))^2

benfordMeanD4 <- mean(benfordVector(4))
benfordVarofD4 <- sd(benfordVector(4))^2

X1df.D1 <- x*(((mean(D1)-benfordMeanD1)^2)/benfordVarofD1) # chi-square test
with 1df for first digit
pchisq(X1df.D1, df = 1, lower.tail = FALSE) # p value for chi-square 1df

## [1] 1.627311e-06

X1df.D2 <- (x-sum(is.na(D2)))*(((mean(D2,na.rm=T)-benfordMeanD2)^2)/benfordVa
rofD2) # chi-square test with 1df for second digit
pchisq(X1df.D2, df = 1, lower.tail = FALSE) # p value for chi-square 1df

## [1] 0.05786063

X1df.D3 <- (x-sum(is.na(D3)))*(((mean(D3,na.rm=T)-benfordMeanD3)^2)/benfordVa
rofD3) # chi-square test with 1df for third digit
pchisq(X1df.D3, df = 1, lower.tail = FALSE) # p value for chi-square 1df

## [1] 0.4271015

############################################################################
##

#One-tailed mean test for first digit

library(BSDA)

## Loading required package: lattice
```

```
##
## Attaching package: 'BSDA'

## The following object is masked from 'package:datasets':
##
##     Orange

D1ztest <- z.test(D1,  alternative = "greater", mu = benfordMeanD1, sigma.x =
benfordVarofD1)
D1ztest
```

```
##
##  One-sample z-Test
##
## data:  D1
## z = 1.9485, p-value = 0.02568
## alternative hypothesis: true mean is greater than 3.43973
## 95 percent confidence interval:
##  3.454212       NA
## sample estimates:
## mean of x
##  3.532655
```

Searches and methods

Appropriate RSP papers

N.B. Sorted first on column for data which is appropriate and then in ascending order on column for overall weighting

Search result	Publication title	Appropriacy	Why not	Boldness test datapoints	Artificial cutoff	Datapoints weighting	Cutoff weighting	Overall weighting
30	Life-history trade-offs: are they linked to personality in a precocial mammal (Cavia aperea)?	appropriate		203	3600	3	2	5
4	Express yourself: bold individuals induce enhanced morphological defences	appropriate		320	1200	2	3	5
25	Learned parasite avoidance is driven by host personality and resistance to infection in a fish–trematode interaction	appropriate		108	10800	5	1	6
12	Personality composition determines social learning pathways within shoaling fish	appropriate		1706	180	1	7	8
28	Personality, sperm traits and a test for their combined dependence on male condition in guppies	appropriate		90	600	6	4	10
1	Regulation between personality traits: individual social tendencies modulate whether boldness and leadership are correlated	appropriate		160	300	4	6	10
2	Personality and the collective: bold homing pigeons occupy higher leadership ranks in flocks	appropriate		45	600	7	5	12
3	Individual quality and personality: bolder males are less fecund in the hermit crab Pagurus bernhardus	not appropriate	unstandardised startle response durations not provided					
5	Individual boldness is linked with protective shell shape in aquatic snails	not appropriate	no data can be found in the folder on dryad					
6	The bold and the sperm: positive association between boldness and sperm number in the guppy	not appropriate	experimental design and artificial maximum unclear					
7	Ambidextrous ungulates have more flexible behaviour, bolder personalities and migrate less	not appropriate	personality data is not clear and does not span more than one order of magnitude					
8	BOLD and its connection to dopamine release in human striatum: a cross-cohort comparison	not appropriate	not associated with personality					
9	Senescence rates and late adulthood reproductive success are strongly influenced by personality in a long-lived seabird	not appropriate	personality assigned using human derived scale					
10	Interactions between cleaner-birds and ungulates are personality dependent	not appropriate	unstandardised personality data not provided					
11	Links between personality, early natal nutrition and survival of a threatened bird	not appropriate	data in pdf format					
13	Personality composition is more important than group size in determining collective foraging behaviour in the wild	not appropriate	includes author of several papers which were retracted for data irregularities					
14	Individual differences in boldness influence patterns of social interactions and the transmission of cuticular bacteria among group-mates	not appropriate	includes author of several papers which were retracted for data irregularities					
15	Boldness traits, not dominance, predict exploratory flight range and homing behaviour in homing pigeons	not appropriate	insufficient data provided					
16	Social interactions shape individual and collective personality in social spiders	not appropriate	includes author of several papers which were retracted for data irregularities					
17	Host personality predicts cuckoo egg rejection in Daurian redstarts Phoenicurus auroreus	not appropriate	data not clear					
18	Antioxidants safeguard telomeres in bold chicks	not appropriate	data not made available					
19	Who directs group movement? Leader effort vs follower preference in stickleback fish of different personality	not appropriate	unstandardised personality data not provided					
20	Quantum theory with bold operator tensors	not appropriate	not associated with personality					
21	Shy birds play it safe: personality in captivity predicts risk responsiveness during reproduction in the wild	not appropriate	insufficient sample size					
22	Personality-specific carry-over effects on breeding	not appropriate	personality not directly assessed in this study and unstandardised data not provided					

23	Be meek or be bold? A colony-level behavioural syndrome in ants	not appro priate	insufficient data provided					
24	The contribution of additive genetic variation to personality variation: heritability of personality	not appro priate	insufficient data provided					
26	Personality-matching habitat choice, rather than behavioural plasticity, is a likely driver of a phenotype–environment covariance	not appro priate	personality data does not span more than two orders of magnitude					
27	Long-term consistency of personality traits of cattle	not appro priate	data not clear					
29	Sex and boldness explain individual differences in spatial learning in a lizard	not appro priate	insufficient sample size					

RSP search

https://royalsocietypublishing.org/action/doSearch?AllField=personality+OR+boldness+OR+bold&AfterYear=2014&BeforeYear=2022&ContentItemType=research-article&rel=nofollow&startPage=0&pageSize=30

See search results on following pages

THE ROYAL SOCIETY PUBLISHING | All Journals

Anywhere | personality OR boldness OR bold | Advanced Search

Search Filters

Applied filters

research article × | 2014 - 2022 ×

Journal Title

Proceedings of the Royal Society B: Biological Sciences	1079
Royal Society Open Science	996
Philosophical Transactions of the Royal Society B: Biological Sciences	390
Biology Letters	266
Journal of The Royal Society Interface	251

More (5)

Publication Date

2014 | 2022

Author

Boogert, Neeltje J	11
Pruitt, Jonathan N	10
Bshary, Redouan	9
Mace, Ruth	9
Shine, Richard	9

More (20)

1 - 30 out of 3707 results for "personality OR boldness OR bold"

Save search | RSS

Refine search | Sort: Relevance

Export Citations | Track Citations | Add to Favourites

Research articles

Regulation between personality traits: individual social tendencies modulate whether boldness and leadership are correlated

Peggy A. Bevan, Isabella Gosetto, Eliza R. Jenkins, Isobel Barnes and Christos C. Ioannou

Proceedings of the Royal Society B: Biological Sciences | Volume 285, Issue 1880

Published: 13 June 2018 | https://doi.org/10.1098/rspb.2018.0829

Preview Abstract | Abstract | Full text | PDF | References

Abstract

Although consistent behavioural differences between individuals (i.e. personality variation) are now well established in animals, these differences are not always expressed when individuals interact in social groups. This can be key in important social ...

Research article

Personality and the collective: bold homing pigeons occupy higher leadership ranks in flocks

Takao Sasaki, Richard P. Mann, Katherine N. Warren, Tristian Herbert, Tara Wilson and Dora Biro

Philosophical Transactions of the Royal Society B: Biological Sciences | Volume 373, Issue 1746

Published: 26 March 2018 | https://doi.org/10.1098/rstb.2017.0038

Preview Abstract | Abstract | Full text | PDF | References

Abstract

While collective movement is ecologically widespread and conveys numerous benefits on individuals, it also poses a coordination problem: who controls the group's movements? The role that animal 'personalities' play in this question has recently become a ...

Research articles

Individual quality and personality: bolder males are less fecund in the hermit crab *Pagurus bernhardus*

Danielle Bridger, Simon J. Bonner and Mark Briffa

Proceedings of the Royal Society B: Biological Sciences | Volume 282, Issue 1803

Published: 22 March 2015 | https://doi.org/10.1098/rspb.2014.2492

Preview Abstract | Abstract | Full text | PDF | References

Abstract

One explanation for animal personality is that different behavioural types derive from different life-history strategies. Highly productive individuals, with high growth rates and high fecundity, are assumed to live life at a fast pace showing high levels ...

Research articles

Express yourself: bold individuals induce enhanced morphological defences

Kaj Hulthén, Ben B. Chapman, P. Anders Nilsson, Johan Hollander and Christer Brönmark

Proceedings of the Royal Society B: Biological Sciences | Volume 281, Issue 1776

Published: 07 February 2014 | https://doi.org/10.1098/rspb.2013.2703

Preview Abstract | Abstract | Full text | PDF | References

Abstract

Organisms display an impressive array of defence strategies in nature. Inducible defences (changes in morphology and/or behaviour within a prey's lifetime) allow prey to decrease vulnerability to predators and avoid unnecessary costs of expression. Many ...

Animal behaviour

Individual boldness is linked to protective shell shape in aquatic snails

Johan Ahlgren, Ben B. Chapman, P. Anders Nilsson and Christer Brönmark

Biology Letters | Volume 11, Issue 4

Published: 01 April 2015 | https://doi.org/10.1098/rsbl.2015.0029

Preview Abstract | Abstract | Full text | PDF | References

Abstract

The existence of consistent individual differences in behaviour ('animal personality') has been well documented in recent years. However, how such individual variation in behaviour is maintained over evolutionary time is an ongoing conundrum. A well-...

Research articles

The bold and the sperm: positive association between boldness and sperm number in the guppy

Clelia Gasparini, Elizabeth M. Speechley and Giovanni Polverino

Royal Society Open Science | Volume 6, Issue 7

Published: 03 July 2019 | https://doi.org/10.1098/rsos.190474

Preview Abstract

Abstract | Full text | PDF | References

Abstract

Assessing the consequences of personality traits on reproductive success is one of the most important challenges in personality studies and critical to understand the evolutionary implications of behavioural variability among animals. Personality traits ...

Research article

Ambidextrous ungulates have more flexible behaviour, bolder personalities and migrate less

R. Found and C. C. St. Clair

Royal Society Open Science | Volume 4, Issue 2

Published: 01 February 2017 | https://doi.org/10.1098/rsos.160958

Preview Abstract

Abstract | Full text | PDF | References

Abstract

Studies of wildlife have shown consistent individual variation in behavioural plasticity, which affects the rate of adaptation to changing environments. More flexible individuals may thus be more prone to habituation and conflict behaviour, but these ...

Research article

BOLD and its connection to dopamine release in human striatum: a cross-cohort comparison

Terry Lohrenz, Kenneth T. Kishida and P. Read Montague

Philosophical Transactions of the Royal Society B: Biological Sciences | Volume 371, Issue 1705

Published: 05 October 2016 | https://doi.org/10.1098/rstb.2015.0352

Preview Abstract

Abstract | Full text | PDF | References

Abstract

Activity in midbrain dopamine neurons modulates the release of dopamine in terminal structures including the striatum, and controls reward-dependent valuation and choice. This fluctuating release of dopamine is thought to encode reward prediction error (...

Research articles

Senescence rates and late adulthood reproductive success are strongly influenced by personality in a long-lived seabird

Samantha C. Patrick and Henri Weimerskirch

Proceedings of the Royal Society B: Biological Sciences | Volume 282, Issue 1799

Published: 22 January 2015 | https://doi.org/10.1098/rspb.2014.1649

Preview Abstract

Abstract | Full text | PDF | References

Abstract

Studies are increasingly demonstrating that individuals differ in their rate of ageing, and this is postulated to emerge from a trade-off between current and future reproduction. Recent theory predicts a correlation between individual personality and life-...

Research article

Interactions between cleaner-birds and ungulates are personality dependent

Rob Found

Biology Letters | Volume 13, Issue 11

Published: 29 November 2017 | https://doi.org/10.1098/rsbl.2017.0536

Preview Abstract

Abstract | Full text | PDF | References

Abstract

While a growing body of literature explores the ecological implications of consistent individual variation in the behaviour of wildlife, few studies have looked at the reciprocal influences of personality within interspecific interactions, despite the ...

Research articles

Links between personality, early natal nutrition and survival of a threatened bird

Kate M. Richardson, Elizabeth H. Parlato, Leila K. Walker, Kevin A. Parker, John G. Ewen and Doug P. Armstrong

Philosophical Transactions of the Royal Society B: Biological Sciences | Volume 374, Issue 1781

Published: 29 July 2019 | https://doi.org/10.1098/rstb.2019.0373

Preview Abstract

Abstract | Full text | PDF | References

Abstract

There is growing recognition that variation in animal personality traits can influence survival and reproduction rates, and consequently may be important for wildlife population dynamics. Despite this, the integration of personality research into ...

Research articles

Personality composition determines social learning pathways within shoaling fish

Matthew J. Hasenjager, William Hoppitt and Lee A. Dugatkin

Proceedings of the Royal Society B: Biological Sciences | Volume 287, Issue 1936

Published: 07 October 2020 | https://doi.org/10.1098/rspb.2020.1871

Preview Abstract — Abstract | Full text | PDF | References

Abstract

In shaping how individuals explore their environment and interact with others, personality may mediate both individual and social learning. Yet increasing evidence indicates that personality expression is contingent on social context, suggesting that ...

Research articles

Personality composition is more important than group size in determining collective foraging behaviour in the wild

Carl N. Keiser and Jonathan N. Pruitt

Proceedings of the Royal Society B: Biological Sciences | Volume 281, Issue 1796

Published: 07 December 2014 | https://doi.org/10.1098/rspb.2014.1424

Preview Abstract — Abstract | Full text | PDF | References

Abstract

Describing the factors that shape collective behaviour is central to our understanding of animal societies. Countless studies have demonstrated an effect of group size in the emergence of collective behaviours, but comparatively few have accounted for the ...

Research article

Individual differences in boldness influence patterns of social interactions and the transmission of cuticular bacteria among group-mates

Carl N. Keiser, Noa Pinter-Wollman, David A. Augustine, Michael J. Ziemba, Lingran Hao, Jeffrey G. Lawrence and Jonathan N. Pruitt

Proceedings of the Royal Society B: Biological Sciences | Volume 283, Issue 1829

Published: 27 April 2016 | https://doi.org/10.1098/rspb.2016.0457

Preview Abstract — Abstract | Full text | PDF | References

Abstract

Despite the importance of host attributes for the likelihood of associated microbial transmission, individual variation is seldom considered in studies of wildlife disease. Here, we test the influence of host phenotypes on social network structure and the ...

Research article

Social interactions shape individual and collective personality in social spiders

Edmund R. Hunt, Brian Mi, Camila Fernandez, Brandyn M. Wong, Jonathan N. Pruitt and Noa Pinter-Wollman

Proceedings of the Royal Society B: Biological Sciences | Volume 285, Issue 1886

Published: 05 September 2018 | https://doi.org/10.1098/rspb.2018.1366

Preview Abstract — Abstract | Full text | PDF | References

Abstract

The behavioural composition of a group and the dynamics of social interactions can both influence how social animals work collectively. For example, individuals exhibiting certain behavioural tendencies may have a disproportionately large impact on the ...

Research article

Boldness traits, not dominance, predict exploratory flight range and homing behaviour in homing pigeons

Steven J. Portugal, Rhianna L. Ricketts, Jackie Chappell, Craig R. White, Emily L. Shepard and Dora Biro

Philosophical Transactions of the Royal Society B: Biological Sciences | Volume 372, Issue 1727

Published: 03 July 2017 | https://doi.org/10.1098/rstb.2016.0234

Preview Abstract — Abstract | Full text | PDF | References

Abstract

Group living has been proposed to yield benefits that enhance fitness above the level that would be achieved through living as solitary individuals. Dominance hierarchies occur commonly in these social assemblages, and result, by definition, in resources ...

Research articles

Host personality predicts cuckoo egg rejection in Daurian redstarts *Phoenicurus auroreus*

Jinggang Zhang, Peter Santema, Jianqiang Li, Lixing Yang, Wenhong Deng and Bart Kempenaers

Proceedings of the Royal Society B: Biological Sciences | Volume 288, Issue 1953

Published: 16 June 2021 | https://doi.org/10.1098/rspb.2021.0228

Preview Abstract — Abstract | Full text | PDF | References

Abstract

In species that are subject to brood parasitism, individuals often vary in their responses to parasitic eggs, with some rejecting the eggs while others do not. While some factors, such as host age (breeding experience), the degree of egg matching and the ...

Evolutionary developmental biology

Antioxidants safeguard telomeres in bold chicks

Sin-Yeon Kim and Alberto Velando

Biology Letters | Volume 11, Issue 5

Published: 01 May 2015 | https://doi.org/10.1098/rsbl.2015.0211

Preview Abstract Abstract | Full text | PDF | References

Abstract

Telomeres are sensitive to damage induced by oxidative stress, and thus it is expected that dietary antioxidants may support the maintenance of telomere length in animals, particularly those with a fast rate of life (e.g. fast metabolism, activity and ...

Research article

Who directs group movement? Leader effort versus follower preference in stickleback fish of different personality

Shinnosuke Nakayama, Jennifer L. Harcourt, Rufus A. Johnstone and Andrea Manica

Biology Letters | Volume 12, Issue 5

Published: 01 May 2016 | https://doi.org/10.1098/rsbl.2016.0207

Preview Abstract Abstract | Full text | PDF | References

Abstract

During collective movement, bolder individuals often emerge as leaders. Here, we investigate whether this reflects a greater propensity of bold individuals to initiate movement, or a preference for shy individuals to follow a bolder leader. We set up ...

Research article

Quantum theory with bold operator tensors

Lucien Hardy

Philosophical Transactions of the Royal Society A: Mathematical, Physical and Engineering Sciences | Volume 373, Issue 2047

Published: 06 August 2015 | https://doi.org/10.1098/rsta.2014.0239

Preview Abstract Abstract | Full text | PDF | References

Abstract

In this paper, we present a formulation of quantum theory in terms of *bold operator tensors*. A circuit is built up of operations where an operation corresponds to a use of an apparatus. We associate collections of operator tensors (which together comprise ...

Animal behaviour

Shy birds play it safe: personality in captivity predicts risk responsiveness during reproduction in the wild

Ella F. Cole and John L. Quinn

Biology Letters | Volume 10, Issue 5

Published: 01 May 2014 | https://doi.org/10.1098/rsbl.2014.0178

Preview Abstract Abstract | Full text | PDF | References

Abstract

Despite a growing body of evidence linking personality to life-history variation and fitness, the behavioural mechanisms underlying these relationships remain poorly understood. One mechanism thought to play a key role is how individuals respond to risk. ...

Research articles

Personality-specific carry-over effects on breeding

Stephanie M. Harris, Sébastien Descamps, Lynne U. Sneddon, Milena Cairo, Philip Bertrand and Samantha C. Patrick

Proceedings of the Royal Society B: Biological Sciences | Volume 287, Issue 1940

Published: 09 December 2020 | https://doi.org/10.1098/rspb.2020.2381

Preview Abstract Abstract | Full text | PDF | References

Abstract

Carry-over effects describe the phenomenon whereby an animal's previous conditions influence its subsequent performance. Carry-over effects are unlikely to affect individuals uniformly, but the factors modulating their strength are poorly known. Variation ...

Research articles

Be meek or be bold? A colony-level behavioural syndrome in ants

S. E. Bengston and A. Dornhaus

Proceedings of the Royal Society B: Biological Sciences | Volume 281, Issue 1791

Published: 22 September 2014 | https://doi.org/10.1098/rspb.2014.0518

Preview Abstract Abstract | Full text | PDF | References

Abstract

Consistent individual variation in animal behaviour is nearly ubiquitous and has important ecological and evolutionary implications. Additionally, suites of behavioural traits are often correlated, forming behavioural syndromes in both humans and other ...

Research articles

The contribution of additive genetic variation to personality variation: heritability of personality

Ned A. Dochtermann, Tori Schwab and Andrew Sih

Proceedings of the Royal Society B: Biological Sciences | Volume 282, Issue 1798

Published: 07 January 2015 | https://doi.org/10.1098/rspb.2014.2201

Preview Abstract | Abstract | Full text | PDF | References

Abstract

Individual animals frequently exhibit repeatable differences from other members of their population, differences now commonly referred to as 'animal personality'. Personality differences can arise, for example, from differences in permanent environmental ...

Research article

Learned parasite avoidance is driven by host personality and resistance to infection in a fish–trematode interaction

Ines Klemme and Anssi Karvonen

Proceedings of the Royal Society B: Biological Sciences | Volume 283, Issue 1838

Published: 14 September 2016 | https://doi.org/10.1098/rspb.2016.1148

Preview Abstract | Abstract | Full text | PDF | References

Abstract

Cognitive abilities related to the assessment of risk improve survival. While earlier studies have examined the ability of animals to learn to avoid predators, learned parasite avoidance has received little interest. In a series of behavioural trials with ...

Research article

Personality-matching habitat choice, rather than behavioural plasticity, is a likely driver of a phenotype–environment covariance

Benedikt Holtmann, Eduardo S. A. Santos, Carlos E. Lara and Shinichi Nakagawa

Proceedings of the Royal Society B: Biological Sciences | Volume 284, Issue 1864

Published: 04 October 2017 | https://doi.org/10.1098/rspb.2017.0943

Preview Abstract | Abstract | Full text | PDF | References

Abstract

An emerging hypothesis of animal personality posits that animals choose the habitat that best fits their personality, and that the match between habitat and personality can facilitate population differentiation, and eventually speciation. However, ...

Research articles

Long-term consistency of personality traits of cattle

Heather W. Neave, Joao H. C. Costa, Daniel M. Weary and Marina A. G. von Keyserlingk

Royal Society Open Science | Volume 7, Issue 2

Published: 12 February 2020 | https://doi.org/10.1098/rsos.191849

Preview Abstract | Abstract | Full text | PDF | References

Abstract

Personality is often defined as the behaviour of individual animals that is consistent across contexts and over time. Personality traits may become unstable during stages of ontogeny from infancy to adulthood, especially during major periods of ...

Research articles

Personality, sperm traits and a test for their combined dependence on male condition in guppies

Edward Galluccio, Rowan A. Lymbery, Alastair Wilson and Jonathan P. Evans

Royal Society Open Science | Volume 9, Issue 6

Published: 01 June 2022 | https://doi.org/10.1098/rsos.220269

Preview Abstract | Abstract | Full text | PDF | References

Abstract

There is evidence that animal personality can affect sexual selection, with studies reporting that male behavioural types are associated with success during pre- and post-copulatory sexual selection. Given these links between personality and sexual traits,...

Research articles

Sex and boldness explain individual differences in spatial learning in a lizard

Pau Carazo, Daniel W. A. Noble, Dani Chandrasoma and Martin J. Whiting

Proceedings of the Royal Society B: Biological Sciences | Volume 281, Issue 1782

Published: 07 May 2014 | https://doi.org/10.1098/rspb.2013.3275

Preview Abstract | Abstract | Full text | PDF | References

Abstract

Understanding individual differences in cognitive performance is a major challenge to animal behaviour and cognition studies. We used the Eastern water skink (*Eulamprus quoyii*) to examine associations between exploration, boldness and individual ...

Research article

Life-history trade-offs: are they linked to personality in a precocial mammal (*Cavia aperea*)?

Anja Guenther

Biology Letters | Volume 14, Issue 4

Published: 18 April 2018 | https://doi.org/10.1098/rsbl.2018.0086

Preview Abstract

Abstract | Full text | PDF | References

Abstract

Life-history trade-offs are predicted to contribute to the maintenance of personality variation. Individuals with 'fast' lifestyles should develop faster, reproduce earlier and exhibit more risky behaviours. Evidence for such predicted links, however, ...

1 2 3 4 5 6 7 ›

ROYAL SOCIETY JOURNALS
Author benefits
Purchasing information
Submit
Open access membership
Recommend to your library
Contact information
Help

ROYAL SOCIETY PUBLISHING
Our journals
Historical context
Open access
Publishing policies
Permissions
Conferences
Videos
Blog
Manage your account
Terms & conditions
Privacy policy
Cookies

THE ROYAL SOCIETY
About us
Contact us
Fellows
Events
Grants, schemes & awards
Topics & policy
Collections
Venue hire

THE ROYAL SOCIETY

Blog

Copyright © 2022 The Royal Society

RWD search

N.B. Unless otherwise stated, all other search fields left blank

Reason(s) for Retraction:

+Concerns/Issues About Data OR +Falsification/Fabrication of Data OR +Unreliable Data OR +Concerns/Issues About Results

Article Type(s):

Research Article

Publisher:

Royal Society Publishing

Original Paper From Date:

01/01/2014

Original Paper To Date (date of final search):

07/05/2022

See search results on following pages. Studies selected for analysis:

Laskowski KL, Pruitt JN. Evidence of social niche construction: persistent and repeated social interactions generate stronger personalities in a social spider. Proceedings of the Royal Society B: Biological Sciences. 2014 May 22;281(1783):20133166.

Modlmeier AP, Laskowski KL, DeMarco AE, Coleman A, Zhao K, Brittingham HA, McDermott DR, Pruitt JN. Persistent social interactions beget more pronounced personalities in a desert-dwelling social spider. Biology letters. 2014 Aug 31;10(8):20140419.

Login

Version: 1.0.6.0

ISSN 2692-465X

The Retraction Watch Database
Please see this user guide before you get started.
And please consider a U.S. tax-deductible donation to allow us to continue to update and make this resource available.

Author(s): Type to search
Country(s):
Title: Type to search
Reason(s) for Retraction: +Concerns/Issues About Data OR +Falsification/Fabrication of Data OR +Unreliable Data OR +Concerns/Issues About Results
Subject(s):
Article Type(s): Research Article
Journal:
Publisher: Royal Society Publishing
Affiliation(s):
Notes:
URL:

Original Paper — From Date: 01/01/2014 To: 07/05/2022; PubMedID: mm/dd/yyyy; DOI:

Retraction or Other Notices — From Date: To:; PubMedID: mm/dd/yyyy; DOI:; Nature of Notice: Paywalled:

Clear Search

Search

Retraction or Other Notices<br>Title/Subject(s)/Journal — Publisher/Affiliation(s)/Retraction Watch Post URL(s)<br>23 Item(s) Found	Reason(s)	Author(s)	Original Paper Date/PubMedID/DOI	Retraction or Other Notices Date/PubMedID/DOI	Article Type(s)<br>Nature of Notice	Countries<br>Paywalled?<br>Notes
DDP-resistant ovarian cancer cells-derived exosomal microRNA-30a-5p reduces the resistance of ovarian cancer cells to DDP<br>(BLS) Biology - Cancer; (BLS) Biology - Cellular; (BLS) Genetics;<br>*Open Biology* — ***Royal Society Publishing***<br>Department of Obstetrics, Linyi People's Hospital, Linyi 276000, People's Republic of China<br>Department of Surgery, People's Hospital of Luozhuang District, Linyi 276000, People's Republic of China	+Concerns/Issues About Data<br>+Concerns/Issues About Image<br>+Investigation by Journal/Publisher<br>+Investigation by Third Party<br>+Original Data not Provided<br>+Paper Mill<br>+Upgrade/Update of Prior Notice	Rongxia Liu<br>Yiqian Zhang<br>Peiwen Sun<br>Changxin Wang	04/29/2020<br>32343928<br>10.1098/rsob.190173	11/17/2021<br>34784789<br>10.1098/rsob.210329	Research Article<br>Retraction	China<br>No
DDP-resistant ovarian cancer cells-derived exosomal microRNA-30a-5p reduces the resistance of ovarian cancer cells to DDP<br>(BLS) Biology - Cancer; (BLS) Biology - Cellular; (BLS) Genetics;<br>*Open Biology* — ***Royal Society Publishing***<br>Department of Obstetrics, Linyi People's Hospital, Linyi 276000, People's Republic of China<br>Department of Surgery, People's Hospital of Luozhuang District, Linyi 276000, People's Republic of China	+Concerns/Issues About Data<br>+Updated to Retraction	Rongxia Liu<br>Yiqian Zhang<br>Peiwen Sun<br>Changxin Wang	04/29/2020<br>32343928<br>10.1098/rsob.190173	10/21/2021<br>34672203<br>10.1098/rsob.210301	Research Article<br>Expression of concern	China<br>No
Predator macroevolution drives trophic cascades and ecosystem functioning<br>(BLS) Zoology; (ENV) Ecology;<br>*Proceedings of the Royal Society B: Biological Sciences* — ***Royal Society Publishing***<br>Department of Ecology and Evolutionary Biology, University of Toronto, Toronto, Ontario M5S3B2, Canada	+Duplication of Data<br>+Unreliable Data<br>+Upgrade/Update of Prior Notice	Denon Start	07/25/2018<br>30051862<br>10.1098/rspb.2018.0384	10/27/2021<br>34702081<br>10.1098/rspb.2021.2140	Research Article<br>Retraction	Canada<br>No
Predator macroevolution drives trophic cascades and ecosystem functioning<br>(BLS) Zoology; (ENV) Ecology;<br>*Proceedings of the Royal Society B: Biological Sciences* — ***Royal Society Publishing***<br>Department of Ecology and Evolutionary Biology, University of Toronto, Toronto, Ontario M5S3B2, Canada	+Concerns/Issues About Data<br>+Concerns/Issues About Results<br>+Updated to Retraction<br>+Upgrade/Update of Prior Notice	Denon Start	07/25/2018<br>30051862<br>10.1098/rspb.2018.0384	06/23/2021<br>34159798<br>10.1098/rspb.2021.1193	Research Article<br>Expression of concern	Canada<br>No
Predator macroevolution drives trophic cascades and ecosystem functioning<br>(BLS) Zoology; (ENV) Ecology;<br>*Proceedings of the Royal Society B: Biological Sciences* — ***Royal Society Publishing***<br>Department of Ecology and Evolutionary Biology, University of Toronto, Toronto, Ontario M5S3B2, Canada	+Concerns/Issues About Results<br>+Updated to Retraction	Denon Start	07/25/2018<br>30051862<br>10.1098/rspb.2018.0384	03/24/2021<br>33757355<br>10.1098/rspb.2021.0506	Research Article<br>Expression of concern	Canada<br>No
Host–parasitoid evolution in a metacommunity<br>(BLS) Parasitology; (ENV) Ecology;<br>*Proceedings of the Royal Society B: Biological Sciences* — ***Royal Society Publishing***<br>Department of Ecology and Evolutionary Biology, University of Toronto, Toronto, Ontario, Canada M5S 3H3<br>http://retractionwatch.com/2021/05/03/ecologist-who-lost-thesis-awards-earns-expressions-of-concern-after-laptop-stolen/	+Concerns/Issues About Data<br>+Updated to Correction	Denon Start<br>Benjamin Gilbert	05/25/2016<br>27194705<br>10.1098/rspb.2016.0477	03/24/2021<br>33757354<br>10.1098/rspb.2021.0505	Research Article<br>Expression of concern	Canada<br>No
Quantum correlations are weaved by the spinors of the Euclidean primitives<br>(PHY) Mathematics; (PHY) Physics;<br>*Royal Society Open Science* — ***Royal Society Publishing***<br>Einstein Centre for Local-Realistic Physics, 15 Thackley End, Oxford OX2 6LB, UK<br>http://retractionwatch.com/2020/10/22/a-journal-paper-retracted-because-of-questions-about-the-integrity-of-the-mathematics/<br>http://retractionwatch.com/2020/11/25/subtraction-by-addition-a-journal-expresses-concern-again-but-this-time-with-feeling/	+Concerns/Issues About Data<br>+Concerns/Issues About Results<br>+No Further Action<br>+Objections by Third Party<br>+Upgrade/Update of Prior Notice	Joy Christian	05/30/2018<br>29893385<br>10.1098/rsos.180526	10/21/2020<br>33204491<br>10.1098/rsos.201777	Research Article<br>Expression of concern	United Kingdom<br>No

Quantum correlations are weaved by the spinors of the Euclidean primitives<br>(PHY) Mathematics; (PHY) Physics;<br>*Royal Society Open Science* — ***Royal Society Publishing***<br>Einstein Centre for Local-Realistic Physics, 15 Thackley End, Oxford OX2 6LB, UK<br>http://retractionwatch.com/2020/10/22/a-journal-paper-retracted-because-of-questions-about-the-integrity-of-the-mathematics/<br>http://retractionwatch.com/2020/11/25/subtraction-by-addition-a-journal-expresses-concern-again-but-this-time-with-feeling/	+Concerns/Issues About Data<br>+Concerns/Issues About Results	Joy Christian	05/30/2018<br>29893385<br>10.1098/rsos.180526	04/10/2019<br>31183156<br>10.1098/rsos.190530	Research Article<br>Expression of concern	United Kingdom<br>No
Behaviour, morphology and microhabitat use: what drives individual niche variation<br>(BLS) Zoology;<br>*Biology Letters* — ***Royal Society Publishing***<br>Department of Psychology, Neuroscience and Behaviour, McMaster University, Hamilton, Ontario, Canada L8S 4L8	+Duplication of Image<br>+Investigation by Journal/Publisher<br>+Unreliable Data	Raul Costa-Pereira<br>Jonathan N Pruitt	06/05/2019<br>31164064<br>10.1098/rsbl.2019.0266	08/26/2020<br>32843899<br>10.1098/rsbl.2020.0588	Research Article<br>Retraction	Canada<br>No
Behavioural hypervolumes of spider communities predict community performance and disbandment<br>(BLS) Zoology; (SOC) Sociology;<br>*Proceedings of the Royal Society B: Biological Sciences* — ***Royal Society Publishing***<br>Department of Ecology, Evolution, and Marine Biology, University of California, Santa Barbara, CA 93106-9610, USA<br>Department of Environmental Science and Policy, University of California at Davis, One Shields Avenue, Davis, CA 95616, USA<br>Department of Integrative Biology, University of Texas at Austin, Austin, TX 78712, USA<br>Department of Ecology and Evolutionary Biology, University of Arizona, Tucson, AZ, USA<br>Department of Ecology and Evolutionary Biology, University of California, Los Angeles, CA 90095-1606, USA	+Concerns/Issues About Authorship<br>+Concerns/Issues About Data<br>+Upgrade/Update of Prior Notice	Jonathan N Pruitt<br>Daniel I Bolnick<br>Andrew Sih<br>Nicholas DiRienzo<br>Noa Pinter-Wollman	12/14/2016<br>27974515<br>10.1098/rspb.2016.1409	08/19/2020<br>32811321<br>10.1098/rspb.2020.1852	Research Article<br>Correction	United States<br>No
Behavioural hypervolumes of spider communities predict community performance and disbandment<br>(BLS) Zoology; (SOC) Sociology;<br>*Proceedings of the Royal Society B: Biological Sciences* — ***Royal Society Publishing***<br>Department of Ecology, Evolution, and Marine Biology, University of California, Santa Barbara, CA 93106-9610, USA<br>Department of Environmental Science and Policy, University of California at Davis, One Shields Avenue, Davis, CA 95616, USA<br>Department of Integrative Biology, University of Texas at Austin, Austin, TX 78712, USA<br>Department of Ecology and Evolutionary Biology, University of Arizona, Tucson, AZ, USA<br>Department of Ecology and Evolutionary Biology, University of California, Los Angeles, CA 90095-1606, USA	+Concerns/Issues About Results<br>+Investigation by Journal/Publisher	Jonathan N Pruitt<br>Daniel I Bolnick<br>Andrew Sih<br>Nicholas DiRienzo<br>Noa Pinter-Wollman	12/14/2016<br>27974515<br>10.1098/rspb.2016.1409	04/29/2020<br>32345165<br>10.1098/rspb.2020.0850	Research Article<br>Expression of concern	United States<br>No
The Achilles' heel hypothesis: misinformed keystone individuals impair collective learning and reduce group success<br>(SOC) Psychology; (SOC) Sociology;<br>*Proceedings of the Royal Society B: Biological Sciences* — ***Royal Society Publishing***<br>Department of Biological Sciences, University of Pittsburgh, Pittsburgh, PA, USA<br>Department of Ecology, Evolution, and Marine Biology, University of California, Santa Barbara, CA, USA<br>Department of Biological Sciences, East Tennessee State University, Johnson City, TN, USA<br>Department of Ecology and Evolutionary Biology, Princeton University, Princeton, NJ, USA<br>BioCircuits Institute, University of California San Diego, La Jolla, CA, USA	+Concerns/Issues About Results<br>+Investigation by Company/Institution<br>+Unreliable Data<br>+Upgrade/Update of Prior Notice	Jonathan N Pruitt<br>Colin M Wright<br>Carl N Keiser<br>Alex E DeMarco<br>Matthew M Grobis<br>Noa Pinter-Wollman	01/27/2016<br>26817771<br>10.1098/rspb.2015.2888	07/29/2020<br>33043869<br>10.1098/rspb.2020.0255	Research Article<br>Retraction	United States<br>No
The Achilles' heel hypothesis: misinformed keystone individuals impair collective learning and reduce group success<br>(SOC) Psychology; (SOC) Sociology;<br>*Proceedings of the Royal Society B: Biological Sciences* — ***Royal Society Publishing***<br>Department of Biological Sciences, University of Pittsburgh, Pittsburgh, PA, USA<br>Department of Ecology, Evolution, and Marine Biology, University of California, Santa Barbara, CA, USA<br>Department of Biological Sciences, East Tennessee State University, Johnson City, TN, USA<br>Department of Ecology and Evolutionary Biology, Princeton University, Princeton, NJ, USA<br>BioCircuits Institute, University of California San Diego, La Jolla, CA, USA	+Concerns/Issues About Results<br>+Investigation by Company/Institution<br>+Updated to Retraction	Jonathan N Pruitt<br>Colin M Wright<br>Carl N Keiser<br>Alex E DeMarco<br>Matthew M Grobis<br>Noa Pinter-Wollman	01/27/2016<br>26817771<br>10.1098/rspb.2015.2888	04/29/2020<br>32345159<br>10.1098/rspb.2020.0847	Research Article<br>Expression of concern	United States<br>No
Reduced interleukin-38 in non-small cell lung cancer is associated with tumour progression<br>(BLS) Biochemistry; (BLS) Biology - Cancer; (BLS) Biology - Cellular; (HSC) Medicine - Oncology; (HSC) Medicine - Pulmonology;<br>*Open Biology* — ***Royal Society Publishing***<br>Department of Respiration, The 1st Hospital of Shangqiu, Shangqiu 476100, People's Republic of China<br>Department of Respiration, The First Affiliated Hospital of Zhengzhou University, Zhengzhou 450002, People's Republic of China	+Duplication of Article<br>+Unreliable Data<br>+Upgrade/Update of Prior Notice	Feng Wang<br>Weihua Zhang<br>Tianfeng Wu<br>Heying Chu	10/31/2018<br>30381361<br>10.1098/rsob.180132	07/22/2020<br>32692958<br>10.1098/rsob.200204	Research Article<br>Retraction	China<br>No
Reduced interleukin-38 in non-small cell lung cancer is associated with tumour progression<br>(BLS) Biochemistry; (BLS) Biology - Cancer; (BLS) Biology - Cellular; (HSC) Medicine - Oncology; (HSC) Medicine - Pulmonology;<br>*Open Biology* — ***Royal Society Publishing***<br>Department of Respiration, The 1st Hospital of Shangqiu, Shangqiu 476100, People's Republic of China<br>Department of Respiration, The First Affiliated Hospital of Zhengzhou University, Zhengzhou 450002, People's Republic of China	+Concerns/Issues About Data<br>+Investigation by Journal/Publisher<br>+Updated to Retraction	Feng Wang<br>Weihua Zhang<br>Tianfeng Wu<br>Heying Chu	10/31/2018<br>30381361<br>10.1098/rsob.180132	06/10/2020<br>32516547<br>10.1098/rsob.200152	Research Article<br>Expression of concern	China<br>No
Reduced miR-125a-5p level in non-small-cell lung cancer is associated with tumour progression<br>(BLS) Biochemistry; (BLS) Biology - Cancer; (BLS) Biology - Cellular; (BLS) Genetics; (HSC) Medicine - Oncology; (HSC) Medicine - Pulmonology;<br>*Open Biology* — ***Royal Society Publishing***<br>Department of Thoracic Surgery, Cancer Hospital of China Medical University, Shenyang 110042, Liaoning Province, People's Republic of China<br>Department of Thoracic Surgery, Liaoning Cancer Hospital and Institute, Shenyang 110042, Liaoning Province, People's Republic of China<br>Department of Medical Imaging, Cancer Hospital of China Medical University, Shenyang 110042, Liaoning Province, People's Republic of China<br>Department of Medical Imaging, Liaoning Cancer Hospital and Institute, Shenyang 110042, Liaoning Province, People's Republic of China	+Author Unresponsive<br>+Duplication of Article<br>+Investigation by Journal/Publisher<br>+Unreliable Data<br>+Upgrade/Update of Prior Notice	Hongxu Liu<br>Yegang Ma<br>Changhao Liu<br>Pengfei Li<br>Tao Yu	10/10/2018<br>30305431<br>10.1098/rsob.180118	07/22/2020<br>32692957<br>10.1098/rsob.200205	Research Article<br>Retraction	China<br>No
Reduced miR-125a-5p level in non-small-cell lung cancer is associated with tumour progression<br>(BLS) Biochemistry; (BLS) Biology - Cancer; (BLS) Biology - Cellular; (BLS) Genetics; (HSC) Medicine - Oncology; (HSC) Medicine - Pulmonology;<br>*Open Biology* — ***Royal Society Publishing***<br>Department of Thoracic Surgery, Cancer Hospital of China Medical University, Shenyang 110042, Liaoning Province, People's Republic of China<br>Department of Thoracic Surgery, Liaoning Cancer Hospital and Institute, Shenyang 110042, Liaoning Province, People's Republic of China<br>Department of Medical Imaging, Cancer Hospital of China Medical University, Shenyang 110042, Liaoning Province, People's Republic of China<br>Department of Medical Imaging, Liaoning Cancer Hospital and Institute, Shenyang 110042, Liaoning Province, People's Republic of China	+Concerns/Issues About Data<br>+Investigation by Journal/Publisher<br>+Updated to Retraction	Hongxu Liu<br>Yegang Ma<br>Changhao Liu<br>Pengfei Li<br>Tao Yu	10/10/2018<br>30305431<br>10.1098/rsob.180118	06/10/2020<br>32516548<br>10.1098/rsob.200151	Research Article<br>Expression of concern	China<br>No
Personality composition is more important than group size in determining collective foraging behaviour in the wild<br>(SOC) Psychology; (SOC) Sociology;	+Concerns/Issues About Results	Carl N Keiser<br>Jonathan N Pruitt	12/07/2014<br>25320170<br>10.1098/rspb.2014.1424	04/29/2020<br>32345158<br>10.1098/rspb.2020.0848	Research Article<br>Expression of concern	United States<br>No

Proceedings of the Royal Society B: Biological Sciences — ***Royal Society Publishing***<br>Department of Biological Sciences, University of Pittsburgh, 182 Crawford Hall, 4249 Fifth Avenue, Pittsburgh, PA 15260, USA	+Investigation by Journal/Publisher					
The legacy effects of keystone individuals on collective behaviour scale to how long they remain within a group<br>(SOC) Sociology;<br>*Proceedings of the Royal Society B: Biological Sciences* — ***Royal Society Publishing***<br>Department of Biological Sciences, University of Pittsburgh, Pittsburgh, PA 15260, USA<br>BioCircuits Institute, University of California San Diego, La Jolla, CA 92093, USA	+Concerns/Issues About Data<br>+Investigation by Journal/Publisher	Jonathan N Pruitt<br>Noa Pinter-Wollman	09/07/2015<br>26336171<br>10.1098/rspb.2015.1766	04/29/2020<br>32345160<br>10.1098/rspb.2020.0849	Research Article<br>Expression of concern	United States<br>No
Persistent social interactions beget more pronounced personalities in a desert-dwelling social spider<br>(BLS) Zoology;<br>*Biology Letters* — ***Royal Society Publishing***<br>Department of Biological Sciences, University of Pittsburgh, 4249 Fifth Avenue, Pittsburgh, PA 15260, USA<br>Department of Biology and Ecology of Fishes, Leibniz Institute of Freshwater Ecology and Inland Fisheries, Mu''ggelseedamm 310, 12587 Berlin, Germany	+Duplication of Data<br>+Unreliable Data	Andreas P Modlmeier<br>Kate L Laskowski<br>Alex E DeMarco<br>Anna Coleman<br>Katherine Zhao<br>Hayley A Brittingham<br>Donna R McDermott<br>Jonathan N Pruitt	08/01/2014<br>25165452<br>10.1098/rsbl.2014.0419	02/19/2020<br>32072862<br>10.1098/rsbl.2020.0062	Research Article<br>Retraction	Germany<br>United States<br>No
Evidence of social niche construction: persistent and repeated social interactions generate stronger personalities in a social spider<br>(BLS) Zoology; (SOC) Sociology;<br>*Proceedings of the Royal Society B: Biological Sciences* — ***Royal Society Publishing***<br>Department of Biology and Ecology of Fishes, Leibniz-Institute of Freshwater Ecology and Inland Fisheries, Müggelseedamm 310, Berlin 12587, Germany<br>Department of Biological Sciences, University of Pittsburgh, Pittsburgh, PA 15260, USA<br>http://retractionwatch.com/2020/01/29/authors-questioning-papers-at-nearly-two-dozen-journals-in-wake-of-spider-paper-retraction/	+Concerns/Issues About Data<br>+Unreliable Results	Kate L Laskowski<br>Jonathan N Pruitt	05/22/2014<br>24671972<br>10.1098/rspb.2013.3166	01/29/2020<br>31992140<br>10.1098/rspb.2020.0077	Research Article<br>Retraction	Germany<br>United States<br>No
Lionfish predators use flared fin displays to initiate cooperative hunting<br>(BLS) Zoology;<br>*Biology Letters* — ***Royal Society Publishing***<br>ARC Centre of Excellence for Coral Reef Studies and School of Marine and Tropical Biology, James Cook University, Townsville, Queensland 4811, Australia<br>Department of Biomedical Sciences, WCVM, University of Saskatchewan, Saskatoon, Saskatchewan, Canada S7N 5B4<br>Department of Biology, University of Saskatchewan, Saskatoon, Saskatchewan, Canada S7N 5E2<br>http://retractionwatch.com/2018/02/28/journal-investigating-earlier-work-by-author-of-discredited-fish-microplastics-paper/	+Concerns/Issues About Data	Oona M Lonnstedt<br>Maud C O Ferrari<br>Douglas P Chivers	06/01/2014<br>24966203<br>10.1098/rsbl.2014.0281	02/14/2018<br>29445046<br>10.1098/rsbl.2018.0062	Research Article<br>Expression of concern	Australia<br>Canada<br>No
Predation selectively culls medium-sized species from island mammal faunas<br>(BLS) Zoology; (ENV) Ecology;<br>*Biology Letters* — ***Royal Society Publishing***<br>Macroevolution and Macroecology Group, Research School of Biology, Australian National University, Canberra, Australia<br>http://retractionwatch.com/2016/12/27/error-laden-database-kills-paper-extinction-patterns/	+Concerns/Issues About Data<br>+Error in Data	Emily Hanna<br>Marcel Cardillo	04/02/2014<br>24694691<br>10.1098/rsbl.2013.1066	10/28/2015<br>26510678<br>10.1098/rsbl.2015.0821	Research Article<br>Expression of concern	Australia
